# Epidemiological characteristics and pathological changes of primary glomerular diseases

**DOI:** 10.1371/journal.pone.0272237

**Published:** 2022-08-18

**Authors:** Yan Li, XiaoYang Yu, WenJing Zhang, Jia Lv, Ping Lan, ZhiGang Wang, JiPing Sun, LiYi Xie, WanHong Lu, XueLiang Feng, Hongli Jiang, Yali Zhang

**Affiliations:** 1 Nephrology Department, The First Affiliated Hospital of Xi’an Jiaotong University, Xi’an, China; 2 Department of Blood Purification, The First Affiliated Hospital of Xi’an Jiaotong University, Xi’an, China; Chinese Academy of Medical Sciences and Peking Union Medical College, CHINA

## Abstract

**Objective:**

By analyzing the pathological characteristics and clinical data of renal biopsy in our hospital in the past 20 years, to further understand the epidemic characteristics and pathological changes of primary glomerular disease, and to provide regional data for the big data of kidney disease in my country.

**Methods:**

A retrospective analysis of 9448 patients with primary glomerular disease who were hospitalized in our hospital from January 1, 2000 to December 31, 2019, aged 18 years or older, and undergoing renal biopsy. Divided every 5 years into a group, a total of 4 groups (first group 2000.1.1–2004.12.31, second groups 2005.1.1–2009.12.31; third groups 2010.1.1–2014.12.31, fourth groups 2015.1.1–2019.12.31).

**Results:**

① There were more males than females, and male: female vs 1.53:1. The proportion of men in the past five years has increased compared with the previous 15 years. ② Mostly middle-aged, with a median age of 41.39 years old. The age is increasing over time. There are differences between the four groups, *P* <0.001; ③ The most common clinical manifestations are nephrotic syndrome, followed by chronic glomerulonephritis. Occult glomerulonephritis, the proportion of patients with nephrotic syndrome increases over time, first to fourth group (40.08%< 42.64% < 47.08%< 53.69%); ④ The most common pathology type from 2000 to 2009 was mesangial proliferative glomerulonephritis. IgA nephropathy was the most common type from 2010 to 2014, but the proportion of membranous nephropathy increased year by year, and it became the most common pathological type from 2015 to 2019; ⑤ The clinical and pathological manifestations of different genders are different, but there is no statistical difference.

**Conclusion:**

In the past 20 years, the primary glomerular disease is mainly middle-aged. There are more men than women. The most common type of clinical manifestation is nephrotic syndrome. The pathological type is mesangial proliferative glomerulonephritis. Over time, the average age is increasing, and the proportion of patients with renal syndrome is increasing. IgA nephropathy is the most common pathological type from 2010 to 2014, and membranous nephropathy has become the main pathological type in the past 5 years.

## 1. Introduction

Kidney disease is a common and frequently occurring disease in China. Every year, nearly 100 people per million people are life-threatening due to the progression of kidney disease from various causes to end stage renal disease (ESRD) [1–3]. Studies on the treatment and prognosis of kidney disease confirm that early appropriate intervention can delay progression of chronic kidney disease (CKD) and reduce the incidence rate and mortality of CKD. Therefore, clarifying the etiology of kidney disease and taking effective prevention and treatment measures are the premise to reduce the incidence of ESRD.

Due to the wide variety of renal diseases and complex etiology and pathogenesis, the clinical manifestations of renal diseases are not consistent with the histological changes of the kidney, and their treatment schemes and the development results of the disease are different. Leishi Li et al. [4] reported that glomerular disease is the main basic disease leading to ESRD in China, accounting for 54.4%. However, the clinical manifestations of glomerular diseases are diverse. Often one clinical manifestation corresponds to at least three or more pathological types. Similarly, one pathological type corresponds to at least three or more clinical manifestations. In addition, the histopathological changes in different stages of renal disease are also different. For example, lgA nephropathy is pathologically manifested in almost all stages of development from nearly normal renal tissue to most glomerulosclerosis. Therefore, understanding the changes of renal histomorphology provides a scientific basis for clinicians to judge the condition, treat the disease and estimate the prognosis.

In terms of the epidemiology of kidney disease, research data show that the incidence of kidney disease is related to region, race, gender and socio-economic environment [5–8]. At present, in addition to some regional case analyses, large domestic studies are mainly the pathological data analysis of 10594 cases of renal biopsy reported by Huiping Chen and Caihong Zeng et al. [9] from 2000 to 2001 and the epidemiological analysis of renal biopsy data in 22 years. It has been more than 10 years since then. According to the age-standardized mortality rate of global diseases from 1990 to 2013 released by the Global Burden of Disease study (CBD) in 2015, chronic kidney disease (CKD) increased by 36.9%, 19 places higher than 36th in the global cause of death, and chronic kidney disease caused more than 500,000 deaths [1]. Yet the rise of chronic kidney disease has received little global attention. Four years later, the 2019 CBD data report [2] shows that from 1990 to 2016, globally, the incidence of CKD increased by 89%, prevalence increased by 87%, annual disability-adjusted life years (DALYs) increased by 62%, and deaths due to CKD increased by 98%. 63% of the BURDEN of CKD is in low—and middle-income countries. Primary glomerulonephritis (PGN) continues to be a major renal disease, and it is important to understand trends in the regional and national disease spectrum to provide an important basis for future health development goals and indicators.

In recent years, there have been few reports on the major epidemic characteristics and pathological types of primary glomerulonephritis in China. Therefore, this study selected 9448 patients who were hospitalized and diagnosed with primary glomerular disease by renal biopsy from January 1, 2001 to December 31, 2019 in the First Affiliated Hospital of Xi’an Jiao tong University, a key third-class a general hospital in Shaanxi Province, and were over 18 years old. The composition characteristics and variation trend of the spectrum of primary glomerular disease were analyzed to provide regional data for big data of kidney disease in China.

## 2. Methods

### 2.1 General information

Xi ’an, a city in northwest China, has a population of more than 10 million. The hospitalized patients with kidney biopsy and definite diagnosis of PGN from January 1, 2001 to December 31, 2019, aged over 18 years old, were collected and divided into 1 group every 5 years, including 4 groups (Group 1 from January 1, 2001 to December 31, 2004, Group 2 from January 1, 2005 to December 31, 2009; Group 3 from January 1, 2010 to December 31, 2014; Group 4 from January 1, 2015 to December 31, 2019). All study procedures were performed on approval by the Ethics Committee of the First Affiliated Hospital of Xi’an Jiaotong University Medical School (permission no: KYLLSL-2020-220). Verbal informed consent was obtained from the patient or their parents or carers.

### 2.2 Renal biopsy method

The automatic biopsy gun produced by BARD Company in the United States was used to locate the lower pole of the kidney and take the kidney tissue. The perforated kidney tissue was divided into 3 parts and examined by light microscopy, immunofluorescence and electron microscopy respectively (since 2013). Kidney biopsy tissues were embedded in paraffin, sectionized and stained with HE, PAS, PASM-Masson and Masson. Patients suspected of kidney damage caused by special components were stained with Congo red, etc. Immunofluorescence was used to observe the location, intensity and distribution characteristics of IgG, IgA, IgM, C3 and Clq deposition in frozen sections of kidney tissues by direct method. HBSAg, HBCAg, κ light chain and λ light chain staining were added (for positive HBV antibody or antigen and abnormal increase of free light chain in blood).

### 2.3 Clinical diagnosis classification and pathological classification

According to the patient’s symptoms, signs, medical history and laboratory examination results, they were diagnosed as follows: Nephrotic syndrome (NS), chronic glomerulonephritis (CGN), acute nephritis syndrome (AGN), rapidly progressive nephritis syndrome (RPGN), latent glomerulonephritis (it can be caused by various pathological types of primary glomerular diseases, but the pathological changes are mostly mild. Most of the diseases have insidious onset and unknown etiology, and their occurrence may be related to immunity, and the pathogenesis is immune system dysfunction leading to glomerular immune damage. The pathological types of occult glomerulonephritis include focal segmental mesangial hyperplasia, mesangial hyperplasia, early membranous, IgA nephropathy, thin basal membrane nephropathy, and early membranous hyperplasia). (LGN), acute renal insufficiency (ARF), and chronic renal insufficiency (CRF) were divided into seven types.

According to the 1995 WHO glomerular disease tissue classification, PGN pathological types were divided into: Mesangial proliferative glomerulonephritis (MsPGN), IgA nephropathy (IgAN), membranous nephropathy (MN), microlesion (MCD), focal segmental glomerulonephritis (FSGS), membranous proliferative nephritis (MPGN), crescentic nephritis (CREGN), capillary proliferative glomerulonephritis (ENPGN), sclerosing glomerulonephritis (SGN), proliferative sclerosing glomerulonephritis (PSGN).

### 2.4 Observation index

The renal pathological examination results were counted, and the clinical diagnosis and pathological type examination results of renal diseases of different gender, age and year were compared.

### 2.5 Statistical methods

SPSS26.0 statistical software was used for analysis. If the measurement data met the normal distribution and homogeneity of variance, X ± S was used as the expression of two independent samples t test; if not, M (Q1 ~ Q3) was used as the expression of median and quartile spacing, and rank sum test was used. The count data were expressed by percentage, χ^2^ test, and rank data by Wilcoxon rank sum test. *α* = 0.05, *P* < 0.05 was statistically significant.

## 3. Results

### 3.1 Epidemiological characteristics

There were 9448 cases, including 5706 males (60.39%) and 3742 females (39.61%), male: female = 1.53:1. The median age was 40.00 years, with an average of 41.27 years (18–85 years) for males and 41.60 years (18–86 years) for females. The proportion of males in each group was higher than that of females. The proportion of males in group 4 was higher than that in the other 3 groups, which was statistically significant. There was no difference among the other 3 groups, as shown in Table 1.

**Table 1:** Comparison of gender composition of each group.

Group	1	2	3	4	Total
N	1008	1806	3274	3360	9448
Male	595 (59.03)	1052 (58.25)	192 1(58.67)	2138 (63.63) *	5706(60.39)
Female	413 (40.97)	754 (41.75)	1353 (41.33)	1222 (36.37)	3742(39.61)

The results were presented as n (percentages) for categorical variables; Group 1 from January 1, 2001 to December 31, 2004, Group 2 from January 1, 2005 to December 31, 2009; Group 3 from January 1, 2010 to December 31, 2014; Group 4 from January 1, 2015 to December 31, 2019.

*Compared with the other three groups, *P*< 0.05.

The comparison of age between each group showed that age was increasing with the passage of time. The comparison between male and female in the same group showed that there were differences between male and female in the other three groups except the fourth group (*P* = 0.058). The differences between the same sex groups were statistically significant, as shown in Table 2.

**Table 2:** Comparison of the ages of different sexes in each group.

Group	1	2	3	4	Total
**Age**	35 (27~47) *	38 (27~51) *	40 (28~51) *	42 (30~55) *	40 (29~52)
Male	34 (25~47)	37 (25~51)	39 (27~52)	44 (31~56)	40 (28~53)
Female	35 (29~47) ^#^	40 (30~50) ^#^	41 (29~51) ^#^	43 (30~54)	41(30~51)

The results were presented as median (Interquartile range, IQR) for continuous variables; Group 1 from January 1, 2001 to December 31, 2004, Group 2 from January 1, 2005 to December 31, 2009; Group 3 from January 1, 2010 to December 31, 2014; Group 4 from January 1, 2015 to December 31, 2019.

^#^ Compared with men in the same group, *P*< 0.05;

*Compared with the other three groups, *P*< 0.05

### 3.2 Analysis of clinical diagnosis composition between groups

The proportion of nephrotic syndrome was the highest in each group, and the proportion of nephrotic syndrome was higher in group 3 and group 4, while the proportion of chronic glomerulonephritis was lower in the group (*P* < 0.05), and there were statistical differences in occultation glomerulonephritis (*P* < 0.05), as shown in Table 3.

**Table 3:** Comparison of clinical manifestations in each group.

Group	1	2	3	4	Total
N	1008	1806	3274	3360	9448
NS	404 (40.08)	770 (42.64) ^+^	1541 (47.08) *	1804 (53.69) *	4519(47.83)
CGN	363 (36.01) *	760 (42.08)	1509 (46.09) ^#^	1440 (42.86)	4072(43.10)
AGN	5 (0.50)	3 (0.17)	5 (0.15)	3 (0.09)	16(0.17)
RPGN	19 (1.89) ^+^	29 (1.61)	21 (0.64)^-^	35 (1.04)	104(1.10)
LGN	208 (20.64) *	230 (12.74) *	164 (5.01) *	46 (1.37) *	648(6.68)
ARF	9 (0.89)	14 (0.78)	27 (0.64)	26 (0.74)	76(0.80)
CRF	0 (0.00)	0 (0.00)	7 (0.21)	6 (0.18)	13(0.14)

The results were presented as n (percentages) for categorical variables; Group 1 from January 1, 2001 to December 31, 2004, Group 2 from January 1, 2005 to December 31, 2009; Group 3 from January 1, 2010 to December 31, 2014; Group 4 from January 1, 2015 to December 31, 2019.

* Compared with other 3 groups, P < 0.05;

^+^ compared with group 3 and 4, *P <* 0.05;

^#^ compared with group 2 and 4, *P <* 0.05;

^-^ Group 3compared with group 2, *P <* 0.05

Acute nephritis syndrome, acute renal insufficiency and chronic renal insufficiency were not compared due to the small number of cases. Nephrotic syndrome = NS, chronic glomerulonephritis = CGN, acute nephritis syndrome = AGN, rapidly progressive nephritis syndrome = RPGN, octagonal nephritis = LGN, acute renal insufficiency = ARF, chronic renal insufficiency = CRF.

### 3.3 Comparison of clinical diagnosis between different genders in each group

The proportion of male in nephrotic syndrome and chronic nephritis was higher than that of female in each group, and the proportion of male in group 4 was higher than that in the other three groups, which was statistically significant. In group 4, the proportion of female patients with acute nephritis was 62.86%, as shown in Table 4.

**Table 4:** Comparison of clinical manifestations of different genders in each group.

Group	1		2		3		4	
	M	F	M	F	M	F	M	F
N (9448)	595(59.03)	413(40.97)	1052(58.25)	754(41.75)	1921(58.67)	1353(41.33)	2138(63.63)	1222(36.37)
NS (4519)	257(63.61)	147(36.39)	449(53.81)	321(41.69)	942(61.13)	599(38.87)	1166(64.63)	638(35.37) *
CGN (4072)	218(60.05)	145(39.95)	467(61.45)	293(38.55)	868(57.52)	641(42.48)	912(63.33)	528(36.67) ^#^
LGN (648)	99(47.60)	109(52.40)	106(46.09)	124(53.91)	78(47.56)	86(52.44)	25(54.35)	21(45.65)
AGN (16)	3(60.00)	2(40.00)	3(100.0)	0(0.00)	1(20.00)	4(80.00)	3(100.00)	0(0.00)
RPGN (104)	12(63.16)	7(36.84)	19(65.52)	10(34.48)	12(57.14)	9(42.86)	13(37.14)	22(62.86) ^+^
ARF (76)	6(66.67)	3(33.33)	8(57.14)	6(42.86)	16(59.26)	11(40.74)	16(61.54)	10(38.46)
CRF (13)	0(0.00)	0(0.00)	0(0.00)	0(0.00)	4(57.14)	3(42.86)	3(50.00)	3(50.00)

The results were presented as n (percentages) for categorical variables; Group 1 from January 1, 2001 to December 31, 2004, Group 2 from January 1, 2005 to December 31, 2009; Group 3 from January 1, 2010 to December 31, 2014; Group 4 from January 1, 2015 to December 31, 2019.

* Compared with groups 2 and 3, *P*< 0.05;

^#^ Compared with group 3, *P*< 0.05;

^+^ 4 group compared with 2 group, *P*< 0.05

Acute nephritis syndrome, acute renal insufficiency and chronic renal insufficiency were not compared due to the small number of cases. Nephrotic syndrome = NS, chronic glomerulonephritis = CGN, acute nephritis syndrome = AGN, rapidly progressive nephritis syndrome = RPGN, octagonal nephritis = LGN, acute renal insufficiency = ARF, chronic renal insufficiency = CRF.

### 3.4 Analysis of the composition of pathological types between groups

The pathological composition of the four groups was significantly different (χ^2^ = 807.206, *P* = 0.00 < 0.05), it indicates that the pathological composition of the four groups is different. The proportion of MN increased gradually among groups, and reached 37.05% in group 4, accounting for the largest proportion of the group. The ratio between MsPGN and MN groups is the same. The variation of IgAN was small in the first 3 groups, but significantly decreased in the fourth group, which was different from the other 3 groups. There was also a significant increase in MCD in group 4. The most common pathological diagnoses in each group were IgA nephropathy, mesangial proliferative nephritis and membranous nephropathy; MsPGN was the most common in group 1 and group 2; IgAN was the most common in group 3, and the proportion of membranous nephropathy increased year by year, and in group 4, it surpassed IgAN and ranked first, as shown in Table 5.

**Table 5:** Comparison of pathological types in each group.

Group	N	1	2	3	4
N	9448	1008	1806	3274	3360
MsPGN	2564(27.14)	378(37.50)	607(33.61)	920(28.10) *	659(19.61) *
IgAN	2962(31.35)	342(33.93)	589(32.61)	1059(32.35) ^-^	972(28.93) *
MN	2500(26.46)	114(11.31) *	340(18.83) *	801(24.47) *	1245(37.05) *
MPGN	117(1.24)	33(3.27) *	18(1.00)	29(0.89)	37(1.10)
FSGS	477(5.05)	51(5.06)	109(6.04) ^#^	205(6.26) ^-^	112(3.33)
PSGN	283(3.00)	30(2.98)	73(4.04) ^#^	111(3.39) ^-^	69(2.05)
SGN	159(1.68)	37(3.67) ^+^	43(2.38)	65(1.99)	14(0.42) *
MCD	262(2.77)	2(0.20)	6(0.33)	48(0.015) *b	207(6.16) *
CREGN	99(1.05)	15(1.49)	16(0.89)	32(0.98)	36(1.07)
ENPGN	25(0.26)	6(0.60)	5(0.28)	5(0.15)	9(0.27)

The results were presented as n (percentages) for categorical variables; Group 1 from January 1, 2001 to December 31, 2004, Group 2 from January 1, 2005 to December 31, 2009; Group 3 from January 1, 2010 to December 31, 2014; Group 4 from January 1, 2015 to December 31, 2019.

* Compared with other 3 groups, *P* < 0.05;

^+^ Group 1 compared with group 3, *P <* 0.05;

^#^ Group 2 compared with group 4 *P <* 0.05;

^-^ Group 3compared with group 4, *P <* 0.05;

Mesangial proliferative glomerulonephritis = MsPGN, IgA Nephropathy = IgAN, membranous nephropathy = MN, microlesion = MCD, focal segmental glomerulonephritis = FSGS, membranous proliferative nephritis = MPGN, crescent nephritis = CREGN, capillary proliferative nephritis (ENPGN), sclerosing glomerulonephritis (SGN), proliferative sclerosing glomerulonephritis (PSGN); Those that are not statistically significant do not need to be marked.

### 3.5 Comparison of pathological types between different genders in each group

In recent 10 years, female was dominant in CREGN, while male was dominant in other pathological types. Gender ratio in all pathological types, except MsPGN (*P* = 0.00 < 0.05) and MPGN (*P* = 0.02 < 0.05), there was no statistical significance in other 4 time periods, and the rest were shown in Table 6.

**Table 6:** Comparison of pathological types of different genders in each group.

Group	N	1		2		3		4	
		M	F	M	F	M	F	M	F
N	9448	595	413	1052	754	1921	1353	2138	1222
MsPGN	2560(27.10)	202(53.44)	176(46.56)	321(52.88)	286(41.12)	518(56.25)	402(43.74)	415(62.95)	244(37.04) *
IgAN	2962(31.35)	211(61.70)	131(37.97)	350(59.42)	239(40.58)	628(59.30)	431(40.70)	568(58.44)	404(41.56)
MN	2500(26.46)	76(66.67)	38(33.33)	216(63.53)	124(36.47)	466(58.18)	335(41.52)	834(66.99)	411(33.01)
MPGN	117(1.24)	20(60.61)	13(39.39)	9(50.00)	9(50.00)	20(68.97)	9(31.03)	31(83.78)	6(16.22) *
FSGS	477(5.05)	28(54.90)	23(45.10)	57(52.29)	52(47.71)	126(61.46)	79(38.54)	71(63.39)	41(36.61)
PSGN	159(1.68)	25(67.57)	12(32.43)	29(67.44)	14(32.56)	45 (69.23)	20(30.77)	12(85.71)	2(14.29)
SGN	283(3.00)	16(53.33)	14(46.67)	54(73.97)	19(26.03)	74(66.67)	37(33.33)	51(73.91)	18(26.09)
MCD	266(2.82)	2(100.00)	0(0.00)	5(83.33)	1(16.67)	27(58.33)	20(41.67)	135(65.23)	72(34.76)
CREGN	99(1.05)	9(60.00)	6(40.00)	8(50.00)	8(50.00)	15(46.88)	17(53.13)	15(41.67)	21(58.33)
ENPGN	25(0.26)	6(100.00)	0(0.00)	3(60.00)	2(40.00)	2(40.00)	3(60.00)	6(66.67)	3(33.33)

The results were presented as n (percentages) for categorical variables; Group 1 from January 1, 2001 to December 31, 2004, Group 2 from January 1, 2005 to December 31, 2009; Group 3 from January 1, 2010 to December 31, 2014; Group 4 from January 1, 2015 to December 31, 2019.

* Compared with other 3 groups, *P <* 0.05;

Mesangial proliferative glomerulonephritis = MsPGN, IgA Nephropathy = IgAN, membranous nephropathy = MN, microlesion = MCD, focal segmental glomerulonephritis = FSGS, membranous proliferative nephritis = MPGN, crescent nephritis = CREGN, capillary proliferative nephritis (ENPGN), sclerosing glomerulonephritis (SGN), proliferative sclerosing glomerulonephritis (PSGN); Those that are not statistically significant do not need to be marked.

## 4. Discussion

Primary glomerular disease is a common kidney disease in China and the main cause of chronic renal failure [10]. Alwall first underwent percutaneous renal biopsy in 1944 [11]. Since Iversen and Brunn’s 1951 study pointed out that percutaneous kidney biopsy was the most useful tool for the diagnosis of kidney disease [12], percutaneous kidney biopsy has become a widely used diagnostic method all over the world, putting an end to the era when people could only rely on autopsy kidney samples to understand kidney disease. In the following years, the development of immunofluorescence microscopy and electron microscopy established the diagnostic status of this technique. Therefore, renal biopsy has become an important way to understand the histopathology, pathogenesis and classification of renal diseases, and to guide the treatment. The background of this study is the composition characteristics and variation trend of primary glomerular disease spectrum spanning 20 years from the beginning of 2000 to the end of 2019 in the First Affiliated Hospital of Xi’an Jiaotong University, with a total number of 9448 cases. In recent 5 years, it is the kidney disease study with the largest time span and single center sample size in China, following the 36-year kidney disease spectrum released by General Hospital of Nanjing Military Region [13]. It is the first one in Shaanxi province in the field of primary glomerular disease spectrum.

There are many reports about kidney diseases undergoing kidney biopsy all over the world, but primary glomerulonephritis is still one of the main kidney diseases in China due to regional, environmental and genetic factors [11–13]. Previously, IgA nephropathy was the most common disease in Asia [14] and Europe [15], while FSGS was the most common disease in the United States [16] and Brazil [17]. Membranous proliferative glomerulonephritis (MPGN) was the most common pathology in South Africa [18] and membranous nephropathy (MN) was the most common pathology in kidney biopsies in Spain [19] and Australia [20]. Even in Asia, there are differences among countries. FSGS is the most common PGN in India and Pakistan, followed by MCD > MN > IgA nephropathy in India, and MN in Pakistan ranks second [21]. MN is the most common PGN in Iran [22], while IgA nephropathy is still dominant in Kuwait and Singapore [23, 24], while the study in Malaysia suggested that MCD is the most common in PGN, followed by FSGS and IgA nephropathy [25].

According to the data analysis of this research center, from 2000 to 2009, mesangial proliferative lesions (MsPGN) ranked the first place, but IgA followed closely with little difference. From 2010 to 2014, IgA nephropathy (32.35%) exceeded MsPGN (29.48%), and MN24.47% ranked the third place. Since 2015, MN (37.05%) has topped IgA in the number of people, increasing by about 1.5 times compared with the previous year. The proportion of MN in male was higher than that in female in all 4 groups. IgA nephropathy and focal stage sclerosive glomerulonephritis showed no statistical significance among the groups, which was consistent with recent reports in China. IgA nephropathy still ranked first in PGN analysis of large renal biopsy disease spectrum from 2000 to 2016 [26], but MN increased rapidly in all reports. For example, in the retrospective study of 6049 patients with renal biopsy conducted by Beijing Medical University from 2003 to 2012 [27], the incidence of idiopathic MN from 2008 to 2012 (29.35%) was nearly double that from 2003 to 2007 (16.8%). The causes are closely related to age and environmental factors. (1) Aging population: With China’s economic development, aging population has become a prominent social problem. Domestic studies [28] show that MN accounts for an absolute majority of the elderly ≥65 years old in renal biopsy. (2) Environmental factors: China is still a developing country. With economic development, environmental pollution has worsened, especially the air quality is worrying, and the winter haze is serious in many places, especially in Xi ’an and Beijing. In 2015, a domestic multi-center study analyzed the reason why the incidence of MN doubled in 10 years (from 2004 to 2014), suggesting that this phenomenon is positively correlated with air quality index, and long-term exposure to PM2.5 may increase the risk of MN, but the specific mechanism is still unclear [29]. However, MN in developed countries, such as Japan [30], Britain [31] and the United States [18], has a declining trend. (3) The trend of younger onset: Liu Zhihong et al. [24] analyzed the data of The National Clinical Research Center for Kidney Diseases of Nanjing General Hospital from 2003 to 2014, and it was found that the incidence of MN continued to increase among adolescent patients, with the maximum increase in all age groups being 14–24 years old.

In this study, the clinical diagnosis of renal biopsy was mainly nephritic syndrome (40.08%–53.69%), which was higher in males than in females in all groups. This is consistent with most reports [21, 22]. However, in the reports of Japan in 2013 [32], the most common clinical diagnosis was chronic nephrotic syndrome (chronic nephritis syndrome refers to the basic clinical manifestations of proteinuria, hematuria, hypertension, and varying degrees of renal dysfunction) (55.4% and 50.0% in 2009 and 2010 respectively), followed by nephrotic syndrome (22.4% and 27.0%). This phenomenon may be related to the different diagnostic criteria of different kidney manifestations in different countries. For example, abnormal urine test was reported as the main indication in Italy [33].

Although the sample size of this study is large, it is still a single-center study and has certain limitations. For example, the proportion of MCD in this study is low (0.06%-2.8%), which is different from other large studies in China (11.33%-18.68%) [15], which may be related to the fact that the diagnosis of MCD mainly depends on electron microscopy. However, the economic development in northwest China is slower than that in south China, and some patients did not receive electron microscopy due to economic reasons. Light microscopy may lead to MCD being described as mesangial proliferative disease (MsPGN). Since the development of electron microscopy in our hospital in 2013, the number of MCD cases has increased significantly, so we considered that the proportion of MCD was underestimated in our study. As a single-center observational study, it can only observe the macroscopic changes of kidney disease, and cannot thoroughly and comprehensively explain the underlying causes. Therefore, basic research is needed to explore.

## 5. Conclusion

To sum up, nearly 20 years of primary glomerular disease mainly middle-aged, more men than women, the most common clinical type of nephrotic syndrome, pathological types of mesangial proliferative glomerulonephritis, over time, the average onset age on the rise, kidney syndrome were increased, the proportion of pathological types in the majority with IgA nephropathy in 2010–2014, Membranous nephropathy became the main pathologic type in recent 5 years.

## Supporting information

S1 File (XLSX)
